# Treatment and outcome of COVID-19 patients in a specialized hospital during the third wave: advance of age and increased mortality compared with the first/second waves

**DOI:** 10.1186/s40981-021-00489-x

**Published:** 2021-12-14

**Authors:** Yutaka Oda, Motoko Shimada, Satoshi Shiraishi, Osamu Kurai

**Affiliations:** 1 Department of Anesthesiology, Osaka City Juso Hospital, 2-12-27 Nonaka-kita, Yodogawa-ku, Osaka, 532-0034 Japan; 2 Department of Respiratory Medicine, Osaka City Juso Hospital, Osaka, Japan; 3 Department of Gastroenterology and Hepatology, Osaka City Juso Hospital, Osaka, Japan

**Keywords:** COVID-19, Pandemic, Specialized hospital, Third wave

## Abstract

**Purpose:**

To elucidate the clinical course of patients with coronavirus disease 2019 (COVID-19) treated at a specialized hospital mainly for those with mild and moderate severity during the third wave, and to compare that with the first and second (1st/2nd) waves.

**Methods:**

We retrospectively reviewed the severity on admission, treatment, and outcome of a total of 581 patients from September, 2020, to March, 2021, and examined the risk factors for deterioration of respiratory condition, defined as requiring oxygen ≥ 7 L/min for 12 h.

**Results:**

The median age was 78 (interquartile range 62−83) years, older than in the 1st/2nd waves (53 years), and 50% of the patients was male. The number of patients classified as mild (peripheral oxygen saturation (SpO_2_) ≥ 96%), moderate I, II, and severe (requiring admission to the ICU or mechanical ventilation) was 121, 324, 132, and 4, respectively. Favipiravir, ciclesonide, dexamethasone, and/or heparin were administered for treatment. Respiratory condition recovered in 496 (85%) patients. It worsened in 81 patients (14%); 51 (9%) of whom were transferred to tertiary hospitals and 30 (5%) died. Mortality rate increased by fivefold compared during the 1st/2nd waves. Age, male sex, increased body mass index, and C-reactive protein (CRP) on admission were responsible for worsening of the respiratory condition.

**Conclusion:**

Patients were older in the third wave compared with the 1st/2nd waves. Respiratory condition recovered in 85%; whereas 5% of the patients died. Old age, male sex, increased body mass index, and CRP would be responsible for worsening of the respiratory condition.

## Introduction

Coronavirus disease 2019 (COVID-19) is a global pandemic, nearly 240 million cases and 4.9 million deaths have been reported until October 2021 [1]. Although the mortality rate of patients with COVID-19 has been approximately 1.1% in Japan and lower than the world average [1], aging of the general population and slow progress of vaccination at the study period are potential risk factors for its rapid prevalence [2].

Our hospital received more than 1600 patients as a specialized hospital for COVID-19 since May, 2020. After publication of our report regarding the first and the second waves of COVID-19 outbreak before September, 2020 [3], there have been three larger waves [4], characterized by the predominance of variants of severe acute respiratory distress coronavirus 2 (SARS-CoV-2) with higher transmissibility than the pre-existing one, and an increase of the number of elderly patients [4, 5], which made the medical system in crisis [6–8]. Although epidemiological information on the COVID-19 outbreak has been disseminated by the government as well as by each prefecture in Japan [4, 9], there is a paucity of data from the viewpoint of hospitals [3, 10, 11], which will provide new insight into and a better guidance for managing the admission and transfer of patients during the COVID-19 pandemic. In this study, we retrospectively analyzed the clinical characteristics of patients with confirmed COVID-19 in our hospital in the third wave, elucidated the causes of deterioration of the respiratory condition, and examined the differences with those during the previous outbreaks.

## Methods

This is a retrospective, observational study at Osaka City Juso Hospital, the first hospital in Japan dedicated to COVID-19 patients with a maximum capacity of 70 patients. After obtaining approval from the institutional ethics committee (No. 2-A17, March 2, 2021), all patients who were admitted to our hospital from September 15, 2020, to March 2, 2021, almost corresponding to the third wave of COVID-19 pandemic, were included in this study. Written informed consent from each patient was waived by opting out. Classification of severity and treatment followed the direction by the Ministry of Health, Labour and Welfare [12], where mild, peripheral oxygen saturation (SpO_2_) ≥ 96%, with no respiratory symptoms or coughing only, no shortness of breath; moderate I (patient does not suffer respiratory failure), 93% < SpO_2_ < 96%, with shortness of breath and pneumonia findings; moderate II (patient suffers respiratory failure), SpO_2_ ≤ 93% and oxygen administration is required; severe, requiring admission to the ICU or mechanical ventilation. All data were collected from the electronic hospital information system as reported previously [3], the last follow-up date was April 2021.

### Statistical analysis

No statistical sample size calculation was performed a priori owing to the nature of the study. We defined improvement as a reduction of the severity from moderate I or II on admission to mild. Patients’ condition was also construed as being improved if they satisfy the criteria for discharge as suggested by the Ministry of Health, Labour and Welfare [12]. We defined worsening as SpO_2_ decreasing to < 93% with inhalational oxygen at 7 L/min for > 12 h, which requires transfer patients to other hospitals for intensive treatment.

Statistical analyses were performed by SigmaPlot 12 (Systat Software Inc., San Jose, California, USA). Continuous variables are presented as median (range), if not specified. Categorial variables are presented as frequency (percentage). Categorical data of the severity and outcome among the three age groups (young, < 40; middle-aged, 40−69; elderly, ≥ 70 years); those between the first and second (1st/2nd) and third waves were analyzed by chi-square test or Fisher’s exact text. For univariate comparisons between patients with improved and worsened respiratory condition, between those during the 1st/2nd and third waves, Mann-Whitney’s *U* test was used. Duration of hospitalization among the three age groups was analyzed by Kruskal-Wallis test, followed by Dunn’s multiple comparisons. *P* < 0.05 was regarded as statistically significant. Explanatory factors were included in multiple logistic regression if *p* values were < 0.20 by univariate analysis.

## Results

### Profiles

A total of 581 patients were admitted. Median age was 78 (interquartile range {IQR}, 62−83; range 18−100 years) and 50% was male (Table 1). Median age was significantly higher (*P* < 0.001), and the proportion of male patients was significantly smaller (*P* = 0.04) compared with those in the 1st/2nd waves [3]. The number of patients classified as mild or moderate I severity requiring no supplemental oxygen was 445 (77%, *n* = 121 and 324, respectively) including 20 pregnant women, and the remaining 136 patients were accompanied with moderate II or severe respiratory condition on admission (*n* = 132 and 4, respectively, Tables 1 and 2). The proportion of the number of patients classified as mild and severe condition was decreased (*P* = 0.01 and < 0.001, respectively), while that with moderate I severity was increased (*P* = 0.005) in the present study compared with the 1st/2nd waves. There were significant differences in the severity among the 3 age groups. Severity in middle-aged (40−69 years) and elderly (≥ 69 years) patients were significantly different from that in young (≤ 40 years) patients (*P* < 0.001 for both, Table 2).

**Table 1:** Characteristics of patients with COVID-19 in the 1st/2nd and the 3rd waves

	1st/2nd waves	3rd wave
Number of patients	300	581
Age ([IQR], range)	53 ([33−72], 15−100)	78 ([62−83], 18−100)***
Male/female	171/129 (57/43)	289/292* (50/50)
Severity (mild/moderate I/moderate II/severe)	85/138/61/16 (28/46/20/6)	121/324/132/4 (21*/55**/23/1***)
Elapsed time from onset to hospitalization ([IQR], range)	7 ([4−9], 1−44)	4 ([2−7], 0−32)***
Pre-transportation site (homes/hotels/nursery homes/hospitals)	206/16/16/62 (69/5/5/21)	395/32/82/72 (68/6/14***/12**)
Duration of hospitalization ([IQR], range)	10 ([7−15], 1−85)	11 ([8−15], 1−49)
Prognosis (recovery/unchanged/worsened/died)	276/3/18/3 (92/1/6/1)	496/4/51/30 (85**/1/9/5)

Data are expressed as the number of patients (percentage) or median ([25−75% interquartile range], range). 1st/2nd waves include patients admitted from March 25 to September 14, 2020. 3rd wave includes patients from September 15, 2020, to March 2, 2021. Data during the 1st/2nd waves were from reference no. 3

*p < 0.05, **p < 0.01, ***p < 0.001 compared with the 1st/2nd waves

**Table 2:** Severity on admission, duration of hospitalization, and outcome of the three age groups

_Age_	_Severity on admission_					_Duration of hospitalization (days)_	_Outcome_			
	_Mild_	_M I_	_M II_	_Severe_	_Total (%)_		_Improve_	_Unchange_	_Worsen_	_Died_
_< 40_	_33_	_14_	_2_	_0_	_49 (9%)_	_8 ([6−10], 1−25)_	_45_	_4_	_0_	_0_
_40−69_	_17_	_86_	_42_	_1_	_146 (25%)**_	_10 ([8−13], 1−28)#_	_128_	_0_	_18_	_0§_
_70 ≤_	_71_	_224_	_88_	_3_	_386 (66%)**_	_12 ([9−18], 1−49)##, ‡_	_323_	_0_	_33_	_30§_
_Total (%)_	_121 (21%)_	_324 (56%)_	_132 (22%)_	_4 (1%)_	_581_	_11 ([8−15], 1−49)_	_496 (85%)_	_4 (1%)_	_51 (9%)_	_30 (5%)_

The number of patients is expressed as the absolute value (percentage). The duration of hospitalization is expressed as median days ([25−75% interquartile range], range)

**P < 0.001 compared with the severity of patients < 40 years and of 40−69 years

^#^*P* < 0.01 and ^##^*P* < 0.001 compared with the duration of hospitalization of patients < 40 years

^‡^*P* < 0.001 compared with the duration of hospitalization of patients of 40−69 years

^§^*P* < 0.001 compared with the outcome of patients < 40 years and of 40−69 years

*M I* moderate I, *M II* moderate II

More than 80% of the patients had been staying in their homes or nursery homes before admission to our hospital (Table 1). Among the 72 patients who had been staying in other hospitals, 29 were transferred for treatment of COVID-19 based on the positive results of PCR test; the remaining 43 were for follow-up treatment of COVID-19 after recovering from severe stage in the tertiary hospitals in Osaka. COVID-19 clusters occurred in hospitals and nursery homes, from where 5 and 60 patients, respectively, were transferred to our hospital. During the third wave, more patients were transferred from nursery homes compared during the 1st/2nd waves (16 vs 82, *P* < 0.001). The median duration from the onset of symptoms to admission was 4 (IQR, 2−7; range, 0−32) days, significantly shorter than our previous study (7 days, *P* < 0.001, Table 1). Of the 28 patients who were admitted to our hospital > 14 days after onset of COVID-19 symptoms, 22 were transferred from other hospitals after intensive treatment such as tracheal intubation and mechanical ventilation; 2 were admitted after staying at hotels; 4 were from home accompanied with mild or lack of symptoms.

### Symptoms and laboratory data

Common presenting symptoms on admission were fever ≥ 37.5 °C, cough, fatigue and dyspnea (Table 3). Only 49 (37%) of 132 patients with moderate II severity complained of dyspnea, and the remaining 83 patients (63%) did not. Notably, 227 (39%) patients reported no symptoms on admission. The most common comorbidities were hypertension, followed by diabetes mellitus, cerebral vascular disease, and dementia (Table 3). Chest XP demonstrated abnormal shadows indicative of pneumonia in 368 (63%) patients. Ground-glass opacity and consolidation typical for COVID-19 pneumonia were detected in 427 (76%) of a total of 563 patients underwent chest CT scan.

**Table 3:** Symptoms on admission and comorbidities

Symptoms	
Fever ≥ 37.5 °C	146 (25)
Cough	137 (24)
Fatigue	88 (15)
Dyspnea	81 (14)
Loss of taste	51 (9)
Loss of smell	43 (7)
Comorbidities	
Hypertension	210 (36)
Diabetes mellitus	121 (21)
Cerebral vascular disease without dementia	66 (11)
Dementia	40 (7)
Ischemic heart disease	38 (7)
Chronic obstructive lung disease	19 (3)
Chronic heart failure	13 (2)
Psychiatric disease	12 (2)
Obstructive sleep apnea	4 (1)

Data are expressed as the number of patients (percentage)

### Treatment and outcome

Median duration of hospitalization was 11 (IQR, 8−15; range, 1−49) days, comparable with our previous study (Tables 1 and 2). There were significant differences in the duration of hospitalization among the three age groups (*P* < 0.001, Table 2). It was significantly longer in elderly patients than in middle-aged and young patients (12 vs. 10 and 12 vs. 8 days, *P* < 0.001 for both). It was also significantly longer in middle-aged than in young patients (*P* = 0.009). Favipiravir, ciclesonide, dexamethasone, and heparin were administered in 363 (62%), 259 (45%), 318 (55%), and 277 (48%) patients, respectively. Ciclesonide was no longer used after publication of the negative results of the randomized study examining its efficacy in December, 2020 [13]. Of the 4 patients with severe condition on admission, 2 were transferred from tertiary hospitals after intensive treatment for 1 and 10 days. They were still requiring supplemental oxygen 5 L/min for maintaining SpO_2_ ≥ 93% after extubation. The remaining 2 patients were emergently transported from home with severe hypoxia, and were transferred to tertiary care hospitals on the day of admission.

Respiratory condition improved in 496 (85%) patients (Tables 1 and 2), consists of 118 (95%), 277 (85%), and 101 (77%) with initial classification of severity of mild, moderate I and II respectively. The proportion of the improved patients to the total number of patients was significantly lower than that in the 1st/2nd waves (85% vs 92%, *P* = 0.004). Of those 496 patients, 372 were discharged home; 69 and 55 were discharged to outside hospitals and nursing facilities, respectively, because of difficulties in daily life after discharge. Most of them stayed in hospitals and nursery homes before transferring to our hospital.

There were four young female patients who were discharged with no changes in the respiratory condition. Two patients (21 and 39 years) with mild and moderate I severity, respectively, were admitted to our hospital. COVID-19 infection was detected in their small children later, who were admitted to another hospital. Both patients were transferred to a hospital where their children were admitted. A 29-year-old pregnant woman at 30 weeks of gestation with moderate I severity was admitted. She was transferred to a tertiary care hospital because of persistent cough and fever > 39.0 °C, for possible cesarean section. A 28-year-old woman with schizophrenia was transferred from a hotel for fever. She was not able to stay in the ward because of anxiety disorder, and was discharged. These four patients stayed in our hospital 1−4 days before transferal.

Respiratory condition worsened in 81 (14%) patients (Table 2), 51 of whom were transferred to tertiary hospitals 5 (IQR 2−7; range 1−14) days after admission. Their condition of admission was moderate I, II, or severe (*n* = 27, 21, and 3, respectively), suggesting that respiratory condition rapidly worsened, requiring intensive treatment, even when they did not require oxygen on admission. The remaining 30 patients died on the median day 12 (IQR: 9−18, range 1−37) after withdrawing treatment, all of whom were > 70 (median, 87) years. Mortality rate was fivefold higher than in the 1st/2nd waves (5% vs 1%). Thirteen of them were involved in COVID-19 cluster in nursery homes and transferred directly or via other hospitals to ours. They or their families did not require advanced treatment including tracheal intubation or mechanical ventilation. Severity on admission was mild, moderate I, moderate II, and severe in 1, 18, 10, and 1 patients, respectively. Tracheal intubation, circulatory support, or kidney replacement therapy was not performed in any patients.

Compared with patients whose condition were improved, those with decreased respiratory condition or died were significantly older, predominantly male sex, with larger body mass index (Table 4). However, the proportion with comorbidities such as hypertension, diabetes mellitus, and chronic obstructive pulmonary diseases was similar between the two groups. Compared with patients with improved respiratory condition, for patients with decreased respiratory condition, aspartate aminotransferase (AST), lactate dehydrogenase (LDH), creatinine, and inflammation-related indices such as C-reactive protein (CRP), D-dimer, and ferritin, but not fibrinogen were significantly elevated, lymphocyte counts were significantly decreased on admission. Multivariate analysis revealed that age, male sex, body mass index, and CRP were associated with worsening the respiratory condition during hospitalization (*P* < 0.01 for all, Table 5).

**Table 4:** Univariate analysis of characteristics, comorbidities, and laboratory data between patients with recovered and worsened respiratory condition

	All (*n* = 581)	Improved (*n* = 496)	Worsened and died (*n* = 81)	*P* value
Age (year)	71 (62−83)	78 (60−83)	79 (71−84)	0.02
Sex (male)	289 (50%)	233 (40%)	56 (69%)	< 0.001
Body mass index (kg/m^2^)	23 (21−26)	23 (21−26)	24 (21−27)	0.02
Comorbidities				
Hypertension	210 (36%)	180 (36%)	30 (37%)	0.90
Diabetes mellitus	121 (22%)	104 (21%)	17 (21%)	1.00
Laboratory data				
White blood cell count (× 10^9^/L)	5.4 (4.1−7.1)	5.4 (4.1−7.1)	5.4 (4.2−7.2)	0.84
Lymphocyte (× 10^9^/L)	1.1 (0.8−1.4)	1.1 (0.8−1.5)	0.9 (0.7−1.2)	0.002
Aspartate aminotransferase (U/L)	31 (23−43)	30 (22−41)	39 (28−49)	< 0.001
Alanine aminotransferase (U/L)	22 (14−36)	22 (14−36)	25 (17−37)	0.064
Lactate dehydrogenase (U/L)	234 (195−304)	229 (193−298)	274 (223−372)	< 0.001
Creatinine (mg/dL)	0.76 (0.60−0.98)	0.75 (0.59−0.96)	0.86 (0.68−1.10)	0.004
C-reactive protein (mg/dL)	2.6 (0.5−5.9)	2.2 (0.4−5.3)	5.8 (2.8−8.8)	< 0.001
Fibrinogen (mg/dL)	418 (337−505)	411 (328−505)	433 (373−538)	0.067
D-dimer (μg/dL)	0.8 (0.6−1.4)	0.8 (0.6−1.1)	1.0 (0.8−1.7)	0.003
Ferritin (ng/mL)	366 (176−721)	351 (168−699)	463 (229−1035)	0.004

Data are expressed as the absolute value (percentage) or median (25−75% interquartile range)

Laboratory data were obtained on the day of admission

**Table 5:** Multivariate logistic regression model for worsening the respiratory condition

	Odds ratio (95% CI)	*p* value
Age	1.06 (1.03−1.08)	< 0.001
Male sex	2.53 (1.39−4.61)	0.002
Body mass index	1.10 (1.04−1.18)	0.003
C-reactive protein	1.13 (1.07−1.19)	< 0.001

## Discussion

The total number of patients with confirmed COVID-19 was 9636 during the 7 months before Sep. 14, 2020 (1st/2nd waves), which was increased to 37,623 between September 15 to March 2 (third wave) in Osaka [9]. Accordingly, the number of admitted patients almost doubled compared with that of the 1st/2nd waves despite the similar duration of the pandemic. The most remarkable difference in patients’ profiles from our previous study [3] was the advanced age (78 vs 53 years). The number of patients with mild severity was decreased while moderate I severity was increased, because respiratory condition was worse in elderly than in young patients, and more elderly patients required hospital treatment. On the other hand, young patients with mild respiratory condition would have been ordered to stay home or in the hotels. Notably, there were almost no sex differences in the number of patients admitted to our hospital, despite more male patients than female ones in the whole population [9, 14]. These changes may result from the characteristics that male patients are more vulnerable to respiratory distress, likely to fall into severe condition, and required intensive treatment at tertiary hospitals than female patients [15].

Median duration from the onset of symptoms to admission was 4 days, remarkably shortened from 7 days in our previous study. It was also significantly shorter than 6−7 days reported in a large cohort study in Japan [16], suggesting rapid deterioration of respiratory condition despite prompt admission from the onset of symptoms. Commonly observed symptoms were fever, cough, and fatigue, in consistent with previous studies [3, 10, 16]. Note that more than 60% of patients with moderate II severity did not complain of dyspnea, suggesting the possibility of asymptomatic hypoxia [17] and the importance of routine measurement of SpO2 regardless of the complaints of patients.

Laboratory data with increased transaminase, inflammation markers, and enhanced coagulation function on admission were almost consistent with previous reports [3, 15]. HT and DM were the representative comorbidities. On the other hand, the number of patients with chronic obstructive pulmonary disease was very small, similar to reports in other countries [15, 18]. There were elderly patients with suspected pulmonary emphysema by radiographic examination but without a diagnosis of COPD in the present study, which may be responsible for the small number.

Agents used for treatment was almost the same as reported in our previous study. We used ciclesonide until Jan. 12, 2021, in a total of 259 patients, but not remdesivir (Veklury®) or casirivimab/imdevimab (Ronapreve®) yet. Although respiratory condition improved in 85% of the total number of patients, it worsened even in patients with mild and moderate I severity on admission, which was not observed in our previous study and emphasizes the importance of careful observation of the respiratory condition.

There were no differences in the ratio of the number of patients with hypertension and diabetes mellitus between patients with improved and worsened respiratory condition (Table 4), which were clearly different from other studies [15] but was consistent with our previous study [3], although the precise reason for it is not clear. All patients requiring further treatment (*n* = 51, 9%) were promptly transferred to tertiary hospitals when SpO_2_ decreased below 93% with oxygen inhalation ≥ 7 l/min with face mask for > 12 h if they required further treatment.

Mortality rate was 5% (30/581), fivefold increase compared with the previous study [3], which would result from advanced age compared with our previous study. The number of unvaccinated elderly people, who are prone to aggravate, has increased. Respiratory condition worsened in many elderly patients despite less severity on admission. They were admitted to our hospital, predominantly from nursery homes, because the patients and their families did not want advanced treatment. These backgrounds would be responsible for fivefold increase of the mortality rate. All of them were > 70 years, similar to other hospitals in Japan, where mortality rate is comparable with ours [11]. Mortality rate in the total number of affected patients with COVID-19 in Osaka prefecture was 1.9% (180/9,636) during the 1st/2nd waves, which was increased to 2.5% (943/37,623) during the third wave [9], despite it was unchanged in overall Japan [1]. Although the mortality rate of the general population in Japan was still lower than other countries because of the unknown reason [1], rapid deterioration of the respiratory condition in this patient population should be noted.

Vaccination is a promising method for preventing infection as well as exacerbation of COVID-19, and has been performed all over the world [19]. Implementation of vaccine of COVID-19 in Japan progressed very sluggishly compared with other developed countries, and only < 1% of people received at least one dose of the vaccination of COVID-19 even in March, 2021, at the end of this study period [20]. With the increasing number of elderly people who has completed vaccination following the policy of prioritizing elderly in Japan [2], the age composition of in-hospital patients with COVID-19 might be changed, and more young and middle-aged patients may require admission.

Of importance the B.1.617.2 (delta) variant of SARS-CoV-2 is rapidly prevailing; however, patients affected with it were not observed during the study period. Because effectiveness of vaccination against clinical outcomes with this variant has been limited [21], an increase of the number of patients affected with the variant is predictable. In preparation for upcoming sixth wave of COVID-19 outbreak, improvement of medical environment such as vaccination to all populations, increase of the number of hospital beds are urgent problems. Also, establishment of medical system so as to adequately respond to the requirement of intensive treatment of patients with severe condition, to facilitate rapid transfer of those patients from neighborhood hospitals, and to avoid overwhelming healthcare capacity would contribute to comprehend the prognosis of patients.

There are several limitations in our study. First, we have treated only patients with mild and moderate severity, implying that the data do not represent the epidemiology of the Osaka area. Patients requiring intensive treatment such as tracheal intubation and mechanical ventilation have been transferred to tertiary hospitals and their outcome is not known. In-hospital mortality is influenced according to the severity of the patients who can be taken care of in our institute. Also, the causes of death of the patients in our hospital were predominantly withdrawal because they or their families did not require further treatment when the condition was worsened. Second, details of patients staying homes and hotels, waiting for admission were not examined. Third, the degree of coagulation function or radiographic findings, and its relation with prognosis, has not been evaluated.

In summary, patients admitted to our hospital during the third wave was older compared with those during the 1st/2nd waves. Although the elapsed time from the onset of symptoms to admission was shortened, mortality rate was increased to 5%. Old age, male sex, increased body mass index, and plasma CRP on admission would be responsible for worsening of the respiratory condition.

## Data Availability

The data used in this case report are available from the corresponding author on reasonable request.

## Abbreviations

COVID-19: Coronavirus disease 2019
SARS-CoV-2: Severe acute respiratory distress coronavirus 2
1st/2nd waves: The first and second waves
